# A novel tsRNA-16902 regulating the adipogenic differentiation of human bone marrow mesenchymal stem cells

**DOI:** 10.1186/s13287-020-01882-6

**Published:** 2020-08-24

**Authors:** Tao Wang, Jun Mei, Xingnuan Li, Xiaoyuan Xu, Baicheng Ma, Weidong Li

**Affiliations:** Key Laboratory of System Bio-medicine of Jiangxi Province, Jiujiang University, Jiujiang, 332000 China

**Keywords:** tRNA-derived small RNAs (tsRNAs), Human bone marrow mesenchymal stem cells (hMSCs), Adipogenic differentiation, Retinoic acid receptor γ (RARγ), Smad 2/3 signaling pathway

## Abstract

**Background:**

Transfer RNA-derived small RNAs (tsRNAs) are a recently discovered form of non-coding RNA capable of regulating myriad physiological processes. The role of tsRNAs in hMSC adipogenic differentiation, however, remains incompletely understood. The purpose of this study was to identify the novel tsRNA-16902 as a regulator of hMSC adipogenic differentiation.

**Methods:**

In this study, we conducted transcriptomic sequencing of hMSCs after inducing their adipogenic differentiation, and we were thereby able to clarify the molecular mechanism underlying the role of tsRNA-16902 in this context via a series of molecular biology methods.

**Results:**

When we knocked down tsRNA-16902 expression, this impaired hMSC adipogenic differentiation and associated marker gene expression. Bioinformatics analyses further revealed tsRNA-16902 to target retinoic acid receptor γ (RARγ). Luciferase reporter assays also confirmed the ability of tsRNA-16902 to bind to the RARγ 3′-untranslated region. Consistent with this, RARγ overexpression led to impaired hMSC adipogenesis. Further analyses revealed that Smad2/3 phosphorylation was increased in cells that either overexpressed RARγ or in which tsRNA-16902 had been knocked down. We also assessed the adipogenic differentiation of hMSCs in which tsRNA-16902 was knocked down and at the same time a Smad2/3 inhibitor was added to disrupt Smad2/3 phosphorylation. The adipogenic differentiation of hMSCs in which tsRNA-16902 was knocked down was further enhanced upon the addition of a Smad2/3 signaling inhibitor relative to tsRNA-16902 knockdown alone.

**Conclusions:**

Through a comprehensive profiling analysis of tsRNAs that were differentially expressed in the context of hMSC adipogenic differentiation, we were able to identify tsRNA-16902 as a previously uncharacterized regulator of adipogenesis. tsRNA-16902 is able to regulate hMSC adipogenic differentiation by targeting RARγ via the Smad2/3 signaling pathway. Together, our results may thus highlight novel strategies of value for treating obesity.

## Background

Adipogenesis is a mechanism whereby pre-adipocytes undergo differentiation and maturation into adipocytes capable of mediating fat storage [1]. Dysfunctional adipogenesis can drive obesity and associated metabolic disorders including type II diabetes mellitus and cardiovascular disease [2, 3]. It is therefore important that the adipogenic process be better understood in an effort to help combat these serious illnesses. Human bone marrow mesenchymal stem cells (hMSCs) are self-renewing multipotent stem cells which can differentiate into many different cell types [4], including adipocytes [5], chondrocytes [6], and osteoblasts [7]. Owing to their robust proliferation and multipotency, hMSCs are frequently utilized for in vitro studies of the mechanisms of adipogenic differentiation [8].

There is increasingly strong evidence demonstrating that small non-coding RNAs (sncRNAs) can serve as key regulators of myriad biological processes including cell differentiation, proliferation [9, 10], and stress responses [11]. Studies of sncRNAs including the well-characterized microRNAs (miRNAs) have shown that these molecules can have a strong impact on biological systems through their ability to modulate target gene expression [12]. Further work has highlighted the potential value of such sncRNAs as tools for molecular biology and for therapeutic use as a means of silencing target gene expression in a targeted manner in contexts where suppressing such gene expression may be advantageous [13].

**Fig. 1:**
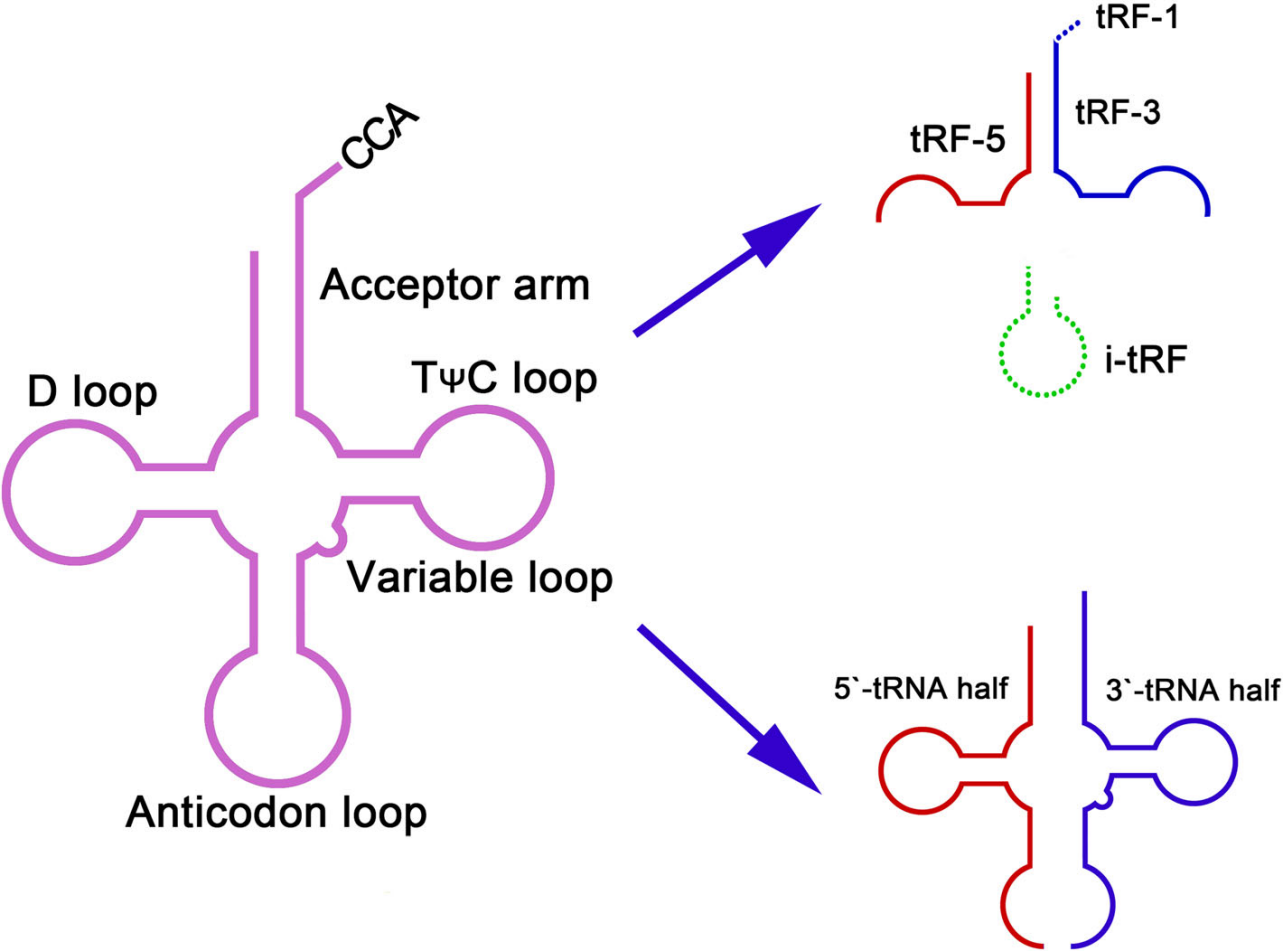
Different types of tRFs are produced from either the pre-tRNA or the mature tRNA. Depending on their origin and composition, these tsRNAs have been grouped into 6 different classes: tRF-5 and tRF-3, i-tRFs, tRF-1, and 5′- and 3′-tRNA halves

Improved sequencing platforms and approaches have recently led researchers to understand that tRNA loci can also give rise to sncRNA molecules, with these tRNA fragments (tRFs), which are also known as tRNA-derived small RNAs (tsRNAs), serving as a recent area of significant research interest [14–17]. These tsRNAs have been grouped into 6 different classes that are defined according to their specific tRNA origins (Fig. 1) [18–20]. Cleavage of tRNAs at the anticodon site by angiogenin yields 5′- and 3′-tRNA halves, whereas mature tRNA cleavage at the D-loop or anticodon step yields tRFs-5, and cleavage at the T-loop or anticodon stem yields tRFs-3. Fragments from the internal portions of mature tRNAs are referred to as internal tRFs (i-tRFs), while tRFs-1 are generated from 3′ end fragments of primary tRNAs.

Given that tsRNA expression has been detected in myriad biological contexts, there has been substantial interest in exploring the relevance of these molecules to normal physiology and to the development of disease. Specific tsRNAs have been found to function through a range of mechanisms in order to suppress oncogenesis [21], regulate cancer cell ribosomal biogenesis [22], influence paternal epigenetic inheritance [23, 24], and facilitate LTR-retrotransposon control [25]. The specific role of tsRNAs in hMSC adipogenic differentiation, however, has yet to be firmly established.

In the present study, we employed a high-throughput sequencing approach in order to identify those tsRNAs that were differentially expressed during the adipogenic differentiation of hMSCs. We then analyzed these tsRNAs in a series of bioinformatics analyses aimed at unraveling their functional importance in the context of adipogenesis. Through this approach, we were able to identify the novel tsRNA-16902 (a 3′-tRNA half) as a tsRNA that regulates hMSC adipogenic differentiation by targeting retinoic acid receptor γ (RARγ) via the Smad 2/3 signaling pathway.

## Materials and methods

### Cell culture and adipogenesis

hMSCs (HUXMA-01001, Cyagen Biosciences, China; cell lot no. of three donors: 150724I31, 160202I31, and 161125R41) were phenotyped via flow cytometry and were confirmed to be ≥ 95% CD73-, CD90-, and CD105-positive, as well as CD11b-, CD19-, CD45-, CD34-, and CD HLA-DR-negative (≤ 5%) as shown in Additional File S1 (Supplementary Files). hMSCs were cultured at 5 × 10^4^ cells/cm^2^ in OriCell hMSC Growth Media (HUXMA-90011; Cyagen Biosciences), supplemented with l-glutamine, 10% FBS (Gibco, Karlsruhe, Germany), and penicillin/streptomycin. Cells were harvested using trypsin-EDTA (Invitrogen, CA, USA) and were passaged until reaching the sixth passage at which time they were used for experimentation.

Adipogenesis was induced by replacing normal hMSC growth media with DMEM, containing 10% FBS, 10% g/mL insulin, 0.5 mM 3-isobutyl-1-methylxanthine, and 0.5 mM dexamethasone after cells had reached confluence [26]. These cells were then cultured in this media for 0, 7, 14, or 21 days after which they underwent high-throughput sequencing analyses.

### Library construction and small RNA-seq

Cellular RNA was extracted using Trizol (Invitrogen), with RNA quality confirmed using an Agilent 2200 machine. Samples that had an RNA integrity (RIN) score > 7.0 were then used for cDNA library preparation with the NEBNext Small RNA Library Prep Kit for Illumina. This RNA was first ligated to provided 3′ and 5′ adapters, after which first strand cDNA synthesis was performed. Index sequences and Illumina sequence adapters were then applied through index PCR, after which the library was purified, assessed for quality control with a Bioanalyzer 2200 instrument (Agilent, CA, USA), and sequences using a HiSeq X-ten platform (Illumina, CA, USA) with 150-bp paired-end reads.

### Small RNA analysis

Low-quality reads and adapter sequences were removed from raw data using Trim Galore, after which all remaining sequences from 12–50 nucleotides long were subjected to sequence alignment with those sequences found in miRBase (http://www.mirbase.org/). Identification of known miRNAs was conducted using BWA after which unmapped reads were aligned to rRNA sequences (https://rnacentral.org/). Next, internally designed tRNA sequence database (using sequences from http://gtrnadb.ucsc.edu/ and https://cm.jefferson.edu/MINTbase/) was used to analyze any remaining unmapped reads. First, intronic sequences were removed after which CCA was added to the end of each tRNA sequence. A total of 50 genomic nucleotides were then added behind these CCA residues, with the resultant mapped reads being identified as potential tsRNAs that were then classified using tRFdb (http://genome.bioch.virginia.edu/trfdb/) and MINTBase (https://cm.jefferson.edu/MINTbase/).

### Differential small RNA expression and target gene prediction

The EB-Seq algorithm [27] was used to identify those tsRNAs that were differentially regulated during adipogenesis based upon *P* value and FDR significance analyses [28], with the cutoff criteria for differentially regulated sncRNAs being (i) fold change > 2 or < 0.5 and (ii) *P* < 0.05, FDR < 0.05. Next, putative tsRNA and miRNA target genes were identified using the miRanda [29] and RNAhybrid tools [30]. A sequencing data analysis flowchart is shown in Fig. S1 (Supplementary Files).

### Lentiviral transduction and hMSC screening

Knockdown of tsRNA-16902 and overexpression of RARγ was conducted using lentiviral particles from Shanghai Genechem Co., Ltd. A short hairpin RNA (shRNA) targeting tsRNA-16902 was designed (target sequence, 5′-TGGTGTCCTTGGAAAAAGGTTTTCATCTCCGGTTTACAA-3′). In addition, a negative control (NC) shRNA was used (sequence, 5′-TTCTCCGAACGTGTCACGT-3′). Lentiviral transduction reactions were conducted by plating 5 × 10^4^ hMSCs/cm^2^ in 6-well plates until 20–30% confluent, after which they were infected using 10 μL of the appropriate lentivirus (1 × 10^8^ infectious units/ml) in complete media supplemented with 5 μg/ml polybrene. After 10 h, this transduction media was removed and fresh media was added, after which the cells were grown for an additional 72 h. Cells were then cultured in 0.5 μg/ml puromycin for 48 h, after which they were screened over a 6-day period with fresh media added every 1–2 days.

At various time points during adipogenesis, Oil Red O staining was used to confirm the presence of lipid droplets within cells. In addition, cells were collected to assess the expression of adipogenic markers and RARγ.

### Oil red O staining

Cells were washed with PBS and were then fixed with 10% formalin for 30 min, after which they were washed using 60% isopropanol prior to staining for 10 min with Oil red O (0.3%; Sigma-Aldrich) while shaking gently. Cells were then washed using distilled water to remove free dye. In addition, free dye was eluted from these cells using 100% isopropanol, after which absorbance at 490 nm was assessed via spectrophotometry [31].

### qRT-PCR

TRIzol (Invitrogen) was used to extract cellular RNA as above, and a cDNA Reverse Transcription Kit (Thermo, CA, USA) was then utilized for cDNA preparation. All qRT-PCR reactions were conducted using a SYBR Premix Ex Taq kit (Toyobo, Osaka, Japan) with an ABI Prism 7500 instrument (Applied Biosystems). The primers used in this study are compiled in Table S1 (Supplementary Files). Relative gene expression was quantified using the 2^−ΔΔCt^ method [32, 33].

### Western blotting

Cells were lysed using RIPA buffer, after which 15 μg of protein per sample was boiled in 5 × SDS sample buffer for 5 min, separated via 10% SDS-PAGE, and transferred to a PVDF membrane (Millipore). Blots were blocked with 5% non-fat milk for 2 h and were probed overnight with the following antibodies: rabbit anti-PPARγ (Abcam 191407), rabbit anti-C/EBPα (Abcam 40764), rabbit anti-FABP4 (Abcam 92501), rabbit anti-RARγ (Abcam 191368), rabbit anti-Smad2/3(Abcam 63672), rabbit anti-p-Smad2/3 (Abcam 63399), and mouse anti-β-actin (1:2,000; Abcam 173838). All rabbit antibodies were diluted 1:1000. Blots were then probed for 1 h with an appropriate secondary HRP-linked antibody (1:5000; CST), after which enhanced chemiluminescence (BeyoECL Plus; Beyotime Institute of Biotechnology) was used for protein visualization.

### Luciferase reporter assay

Briefly, a wild-type (WT) or mutated version of the RARγ 3′-UTR sequence containing this potential tsRNA binding site was cloned into the pGL3 vector downstream of luciferase, after which DNA sequencing was used to confirm construct identity. This vector was then co-transfected into 293T cells with or without a tsRNA-16902 mimic. After 48 h, the luciferase activity in these cells was quantified with Renilla luciferase activity being used for normalization purposes.

### Statistical analysis

SPSS v16.0 (SPSS, IL, USA) was used for statistical testing. Data are given as means ± standard deviation (SD). Data were compared using Student’s *t* tests and one-way ANOVAs, as appropriate, with a two-tailed *P* < 0.05 as the significance threshold.

## Results

### Characterization of the tsRNA expression profile of hMSCs undergoing adipogenesis

**Fig. 2:**
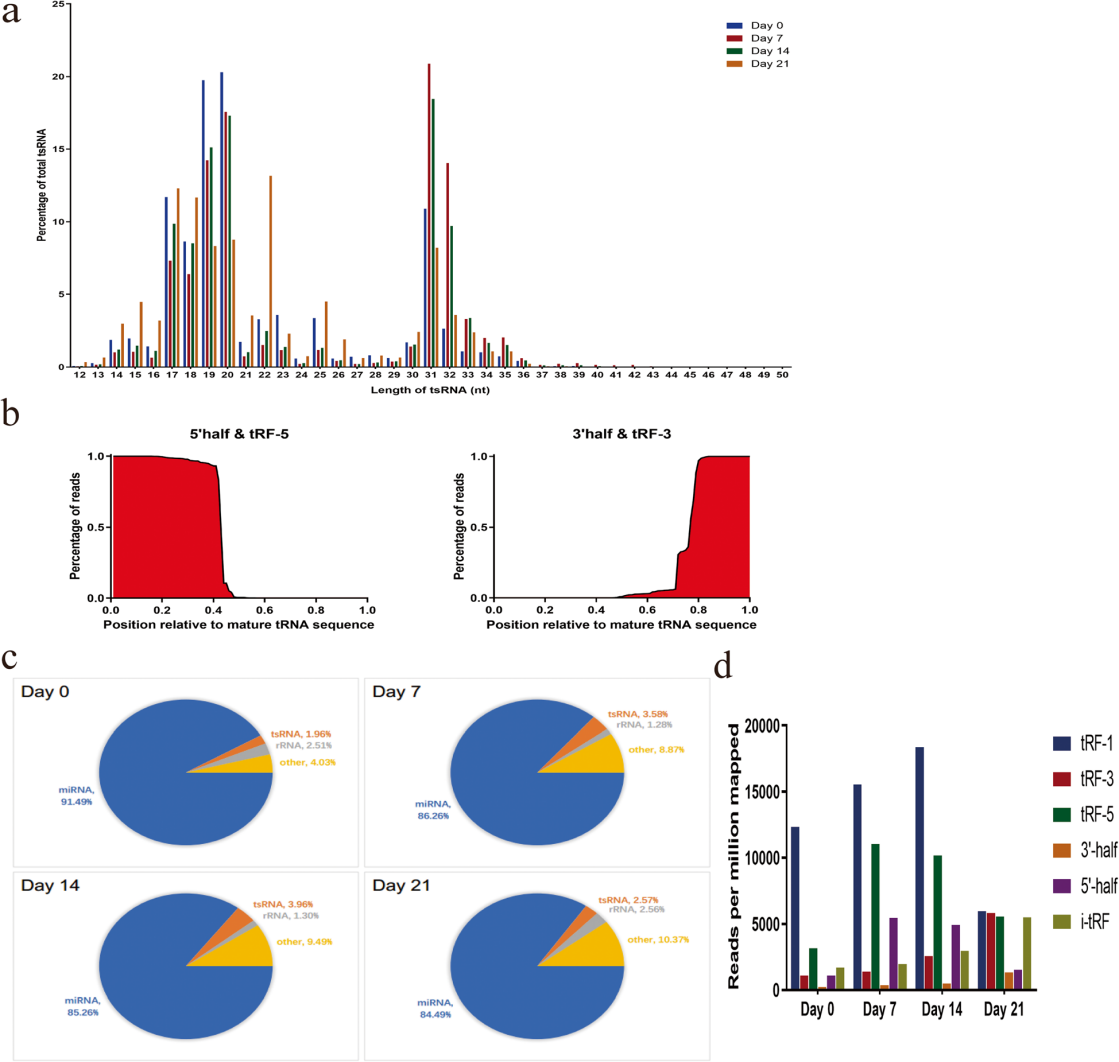
The expression of tsRNAs during hMSC adipogenesis. **a** tsRNA read distributions during adipogenic differentiation, with tsRNA length variant frequencies given as a percentage of total reads. These identified tsRNAs were primarily 17–23 or 30–36 nucleotides long at all time points. **b** 5′-tRF halves and tRF-5 sequences had positions similar to those of parental tRNAs, as was true for the majority of 3′-tRF halves and tRF-3 sequences. **c** The frequencies of tsRNAs during adipogenesis in the overall small RNA-seq, with tsRNA reads provided as a percentage of total reads. The relative frequency of these tsRNAs as a fraction of total non-coding RNAs was significantly increased at all tested time points during adipogenic differentiation. **d** All six primary classes of tRFs were detectable in these samples, with the relative frequencies of individual tRF subtypes varying over the course of adipogenesis and with the most significant increases being evident on days 7 and 14 of this process

We began by assessing which tsRNAs were differentially expressed in hMSCs during the process of adipogenic differentiation by collecting RNA from these cells at days 0, 7, 14, and 21 of adipogenesis and conducting small RNA sequencing using an Illumina HiSeq X ten platform. The GtRNA and piRNA databases were then used to filter out reads from 24 to 33 nucleotides long which were then aligned with those in tRFdb and tRF MINTbase to yield sample tsRNA expression profiles. These identified tsRNAs were primarily from 17 to 23 or 30–36 nucleotides long at all time points (Fig. 2a). In addition, the majority of 5′-tRNA halves, tRFs-5, 3′-tRNA halves, and tRFs-3 had positions similar to those of the parental tRNAs from which they were derived (Fig. 2b).

Relative to baseline, we found that the relative frequency of these tsRNAs as a fraction of total non-coding RNAs was significantly increased at all tested time points during adipogenic differentiation (Fig. 2c). All six primary classes of tRFs were detectable in these samples, with the relative frequencies of individual tRF subtypes varying over the course of adipogenesis and with the most significant increases being evident on days 7 and 14 of this process (Fig. 2d).

### Identification of tsRNAs differentially expressed during adipogenesis

**Fig. 3:**
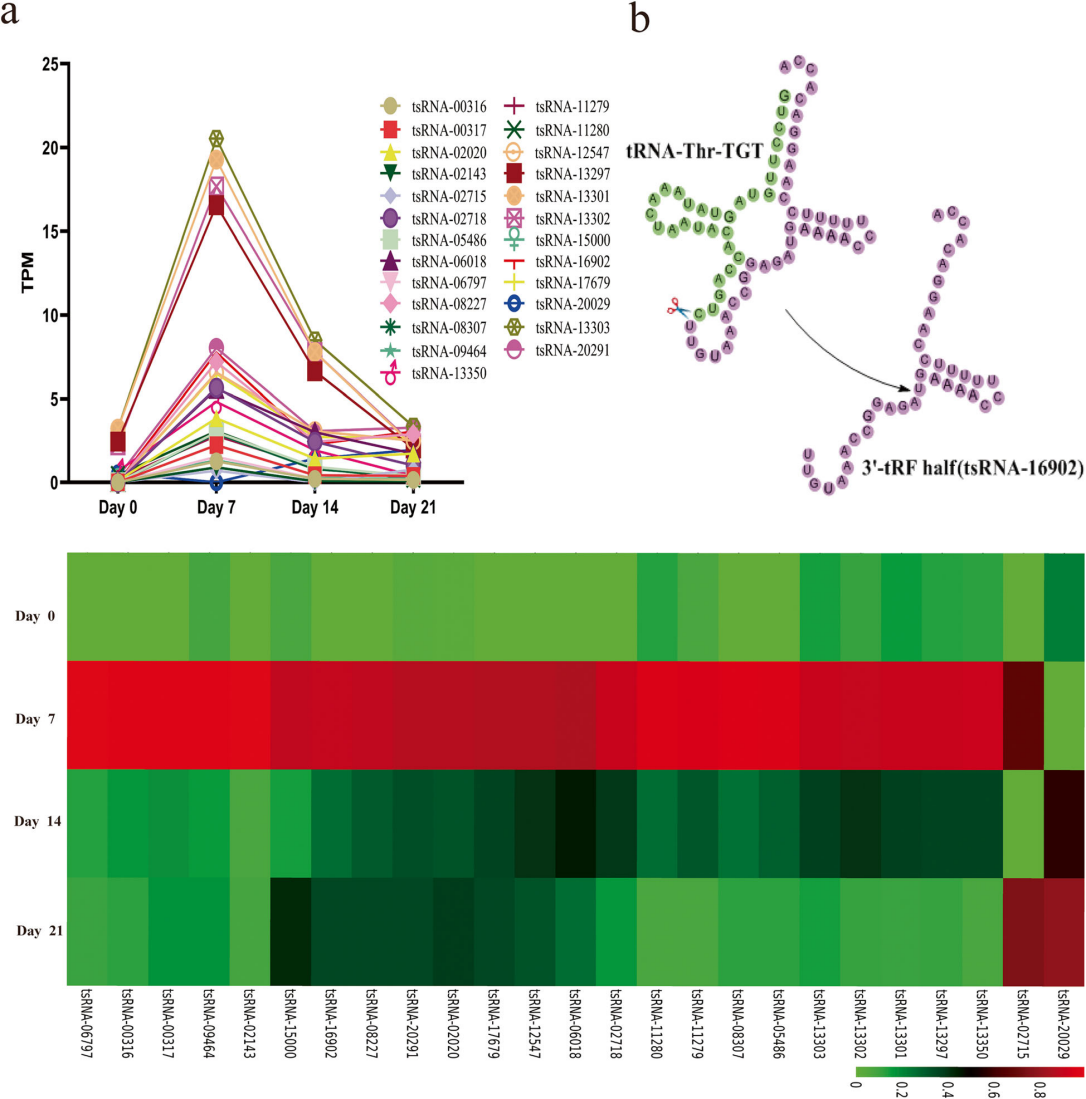
**a** tsRNAs presented differential expression in the context of hMSC adipogenic differentiation. The peak expression of most tsRNAs occurred on day 7 of adipogenesis, and as such, we therefore focused specifically on the comparison of differential tsRNA expression between day 0 and day 7. TPM, transcripts per million reads. **b** In total, we assessed six differentially expressed tsRNAs on day 7, and focused specifically on tsRNA-16902. tRNA-Thr-TGT underwent digestion to yield a corresponding 3′-tRNA half (tsRNA-16902; Database_ID: tRF-39-ZLBS5EOB3ZY61DE2)

We next utilized the EBSeq algorithm in order to identify those tsRNAs that were differentially expressed during hMSC adipogenesis, with Log2FC > 1 and FDR < 0.05 as cut-off criteria in order to identify differentially expressed tsRNAs when comparing samples collected on days 0 and 7, 7 and 14, or 14 and 21. We found that the peak expression of most tsRNAs occurred on day 7 of adipogenesis, and as such, we therefore focused specifically on the comparison of differential tsRNA expression between day 0 and day 7 (Fig. 3a, b). In total, we assessed six differentially expressed tsRNAs at this time point through preliminary experiments and found that the effect of tsRNA-16902 on adipogenic differentiation was more significant as compared to several other tested tsRNAs. Therefore we focused specifically on tsRNA-16902 (3′-tRF half; Database_ID: tRF-39-ZLBS5EOB3ZY61DE2).

### Knockdown of tsRNA-16902 inhibits hMSC adipogenesis

**Fig. 4:**
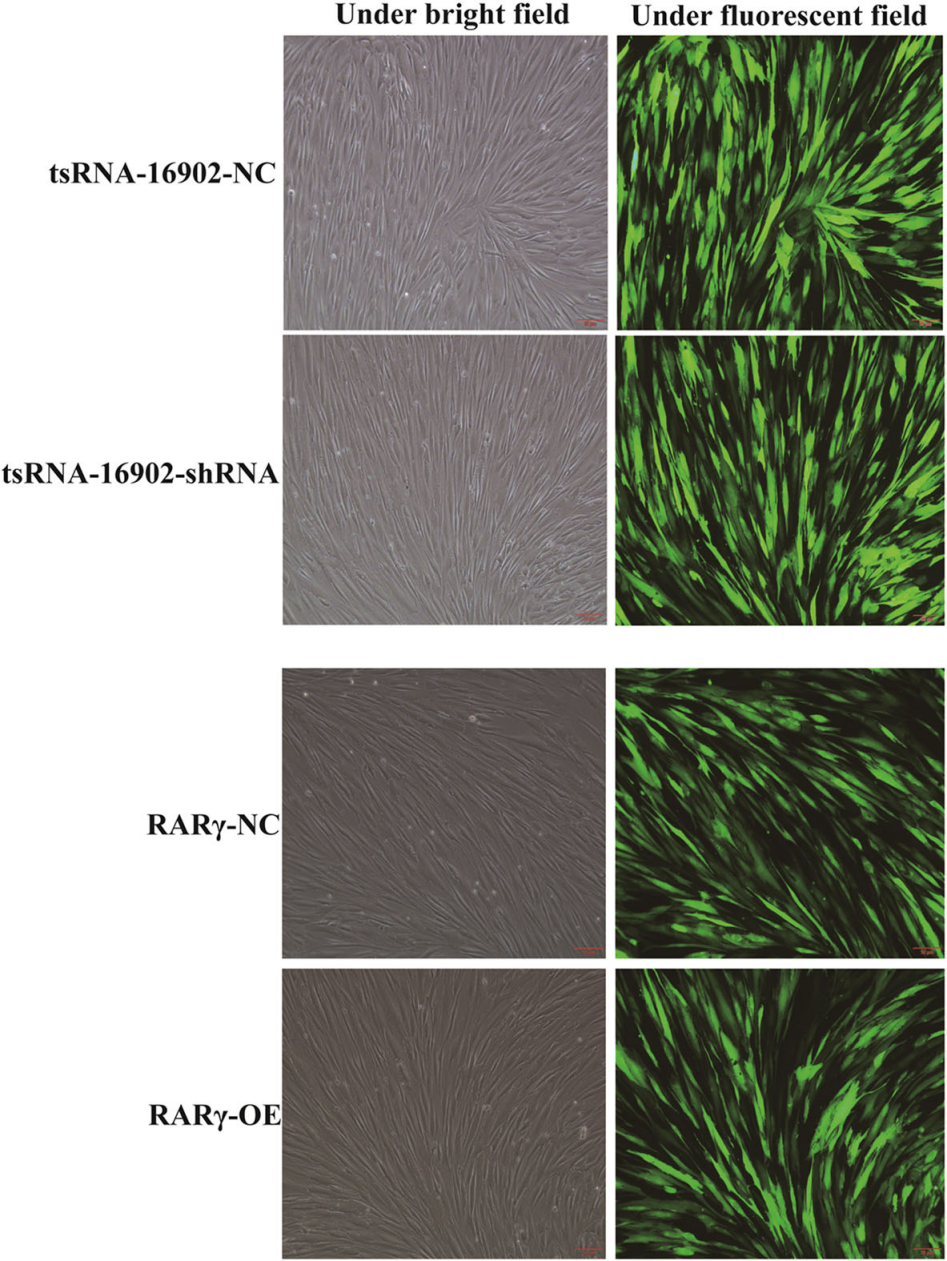
After 6 days of puromycin selection, stably transduced cells that proliferated effectively and expressed GFP were obtained consistent with successful stable transduction. Transduced cells were assessed via light and fluorescent microscopy (10×); scale bar, 50 μm. Note: NC, negative control; OE, overexpression

We next generated hMSCs that were stably transduced to an shRNA specific for tsRNA-16902 or to overexpress RARγ. After 6 days of puromycin selection, stably transduced cells that proliferated effectively and expressed GFP were obtained consistently with successfully stable transduction (Fig. 4).

**Fig. 5:**
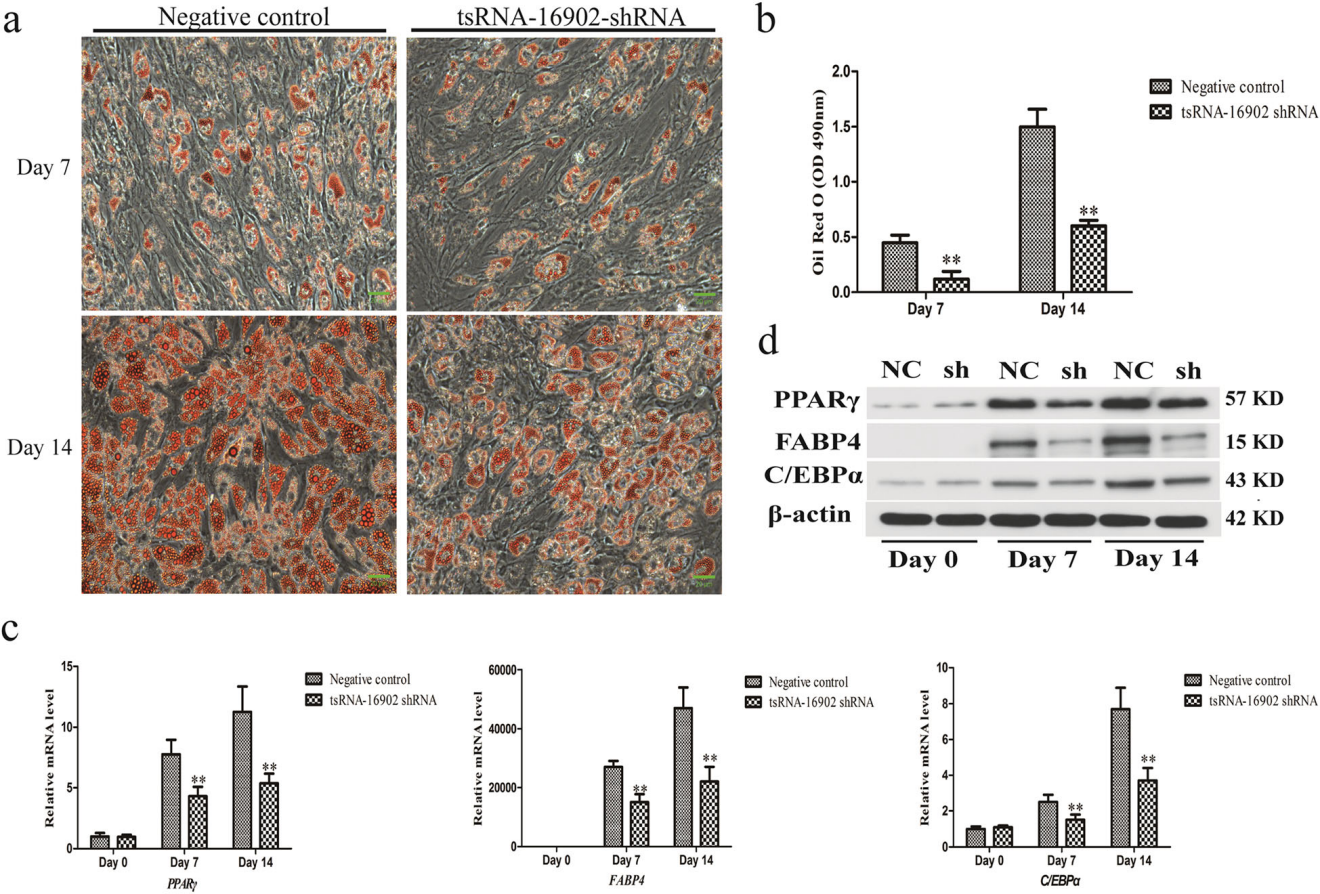
Knocking down tsRNA-16902 leads to impaired the adipogenic differentiation of these hMSCs after 0, 7, and 14 days. **a** hMSCs subjected to oil red O staining were assessed via light microscope at different time points (20×); scale bar, 20 μm. **b** A microplate reader was used in order to quantify oil Red O staining intensity. **c** PPARγ, FABP4, C/EBPα, and RARγ expression was analyzed using qRT-PCR. **d** PPARγ, FABP4, C/EBPα, and RARγ expression was analyzed via Western blotting. Data are means ± SD (*n* = 3). **P < 0.01 vs. negative controls, respectively. Note: NC, negative control; sh, tsRNA-16902 shRNA

We began by exploring the functional importance of tsRNA-16902 in the context of hMSC adipogenesis by knocking down this tsRNA and assessing adipogenic differentiation of these hMSCs after 0, 7, and 14 days. Through Oil Red O staining, we determined that knockdown of this tsRNA significantly suppressed lipid accumulation within these cells consistent with disrupted adipogenesis (Fig. 5a). This was further confirmed when isopropanol elution was used to quantify the intensity of Oil Red O staining in these negative control or tsRNA-16902-knockdown cells (Fig. 5b).

We next examined the impact of tsRNA-16902 knockdown on the expression of the adipogenic markers PPARγ, FABP4, and C/EBPα at the RNA and protein levels. We found that knocking down this tsRNA was associated with significant reductions in the expression of all three of these markers (Fig. 5c, d), thus suggesting that tsRNA-16902 plays a central role in adipogenesis.

### RARγ is a direct tsRNA-16902 target

**Fig. 6:**
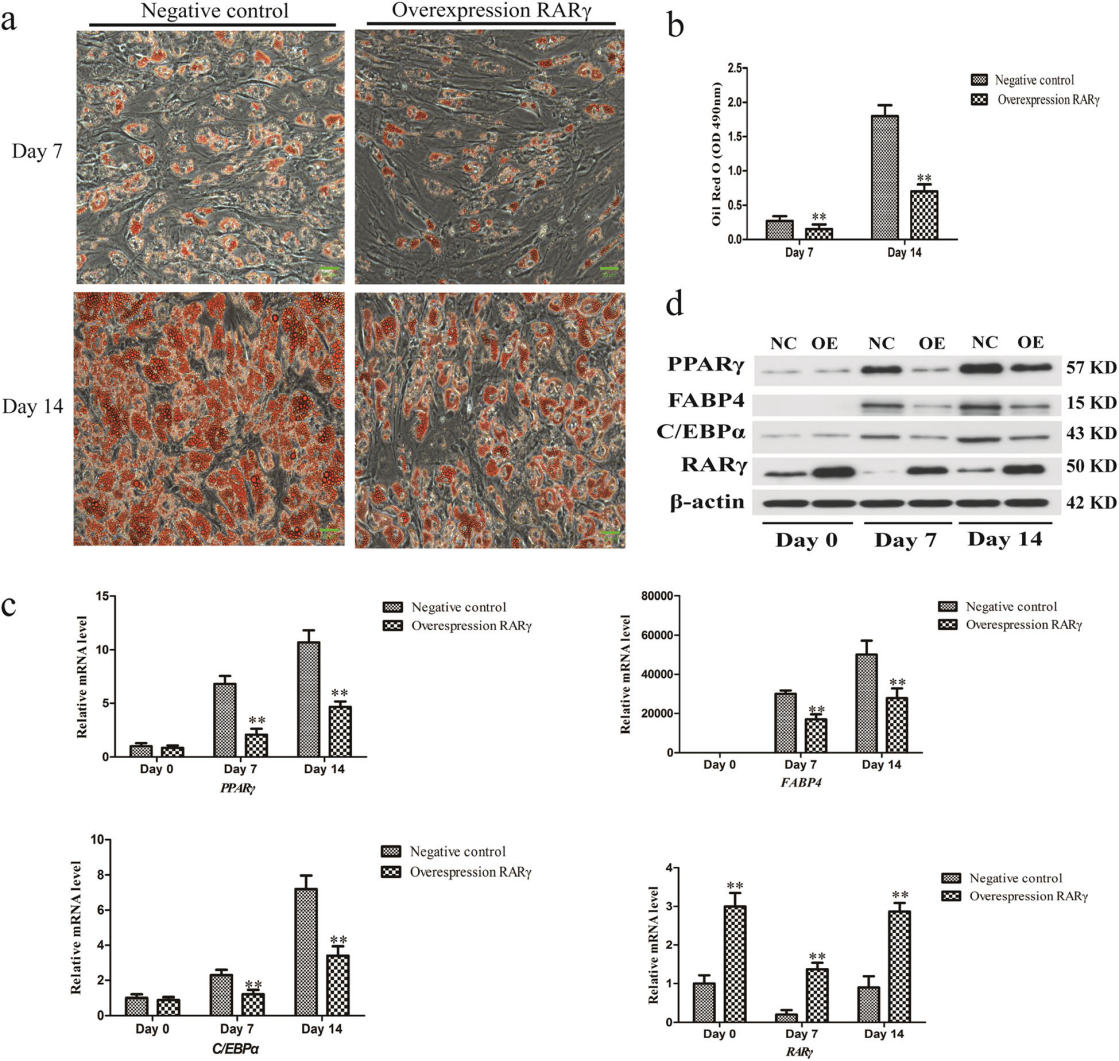
Overexpressing RARγ leads to impaired hMSC adipogenesis on days 0, 7, and 14. **a** hMSCs subjected to oil red O staining were assessed via light microscope at different time points (20×); scale bar, 20 μm. **b** A microplate reader was used in order to quantify oil Red O staining intensity. **c** PPARγ, FABP4, C/EBPα, and RARγexpression was analyzed using qRT-PCR. d PPARγ, FABP4, C/EBPα, and RARγ expression was analyzed via Western blotting. Data are means ± SD (*n* = 3). **P < 0.01 vs. negative controls, respectively. Note: NC, negative control; OE, overexpression RARγ

We next sought to identify potential tsRNA-16902 target genes with the miRanda and RNAhybrid databases, which identified RARγ as a putative target of this tsRNA. We then sought to test the importance of RARγ in the regulation of hMSC adipogenesis by overexpressing this gene in hMSCs. We found that overexpressing RARγ significantly disrupted adipogenesis on days 7 and 14 (Fig. 6a, b), and that this also corresponded to significant reductions in the expression of PPARγ, C/EBPα, and FABP4 (Fig. 6c, d).

**Fig. 7:**
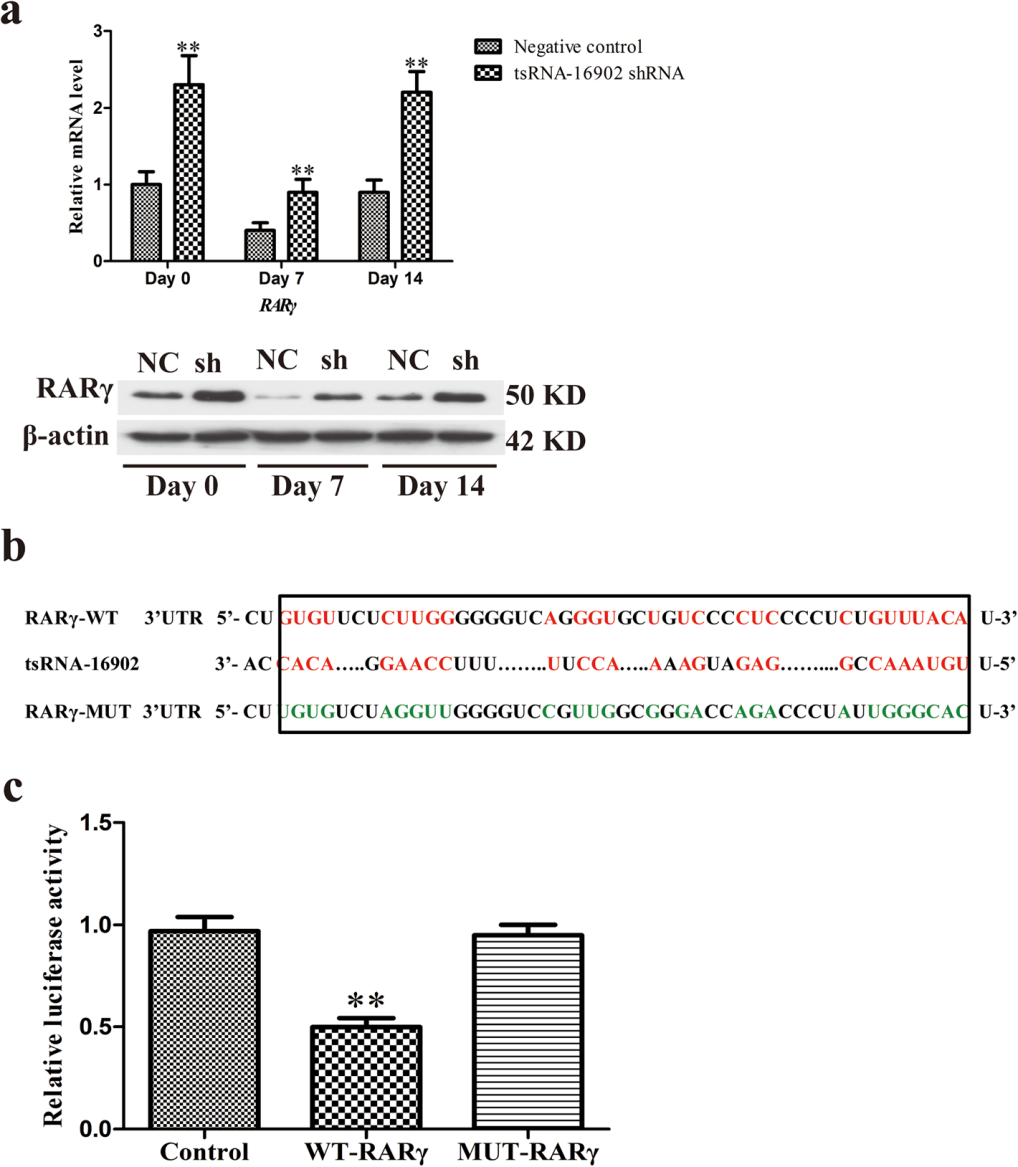
tsRNA-16902 is able to bind to the RARγ 3′-UTR. **a** An analysis of how tsRNA-16902 influenced RARγ expression in the context of hMSC adipogenesis on days 0, 7, and 14. **b** An illustration of the putative site of tsRNA-16902 binding within the 3′-UTR of RARγ, with green used to highlight those nucleotides mutated for a mutant version of this sequence. **c** Luciferase activity assay revealed that tsRNA-16902 transfection into cells resulted in a 50% reduction in luciferase activity for constructs containing a WT but not a mutated version of this RARγ 3′-UTR binding site. Data are means ± SD (*n* = 3). **P < 0.01 vs. negative controls, respectively. Note: NC, negative control; sh, tsRNA-16902 shRNA

We next explored whether tsRNA-16902 was able to directly interact with the RARγ mRNA in order to better characterize the regulatory relationship between these molecules in the context of adipogenesis. We therefore began by assessing how tsRNA-16902 influenced RARγ expression during the adipogenic process (Fig. 7a). We then used TargetScan in order to identify the predicted tsRNA-16902 binding site within the RARγ 3′-UTR (Fig. 7b). To confirm the validity of this binding site, a luciferase reporter assay was then conducted which revealed that tsRNA-16902 transfection into cells resulted in a 50% reduction in luciferase activity for constructs containing a WT but not a mutated version of this RARγ 3′-UTR binding site (Fig. 7c).

### Both tsRNA-16902 knockdown and RARγ overexpression increase Smad2/3 phosphorylation

**Fig. 8:**
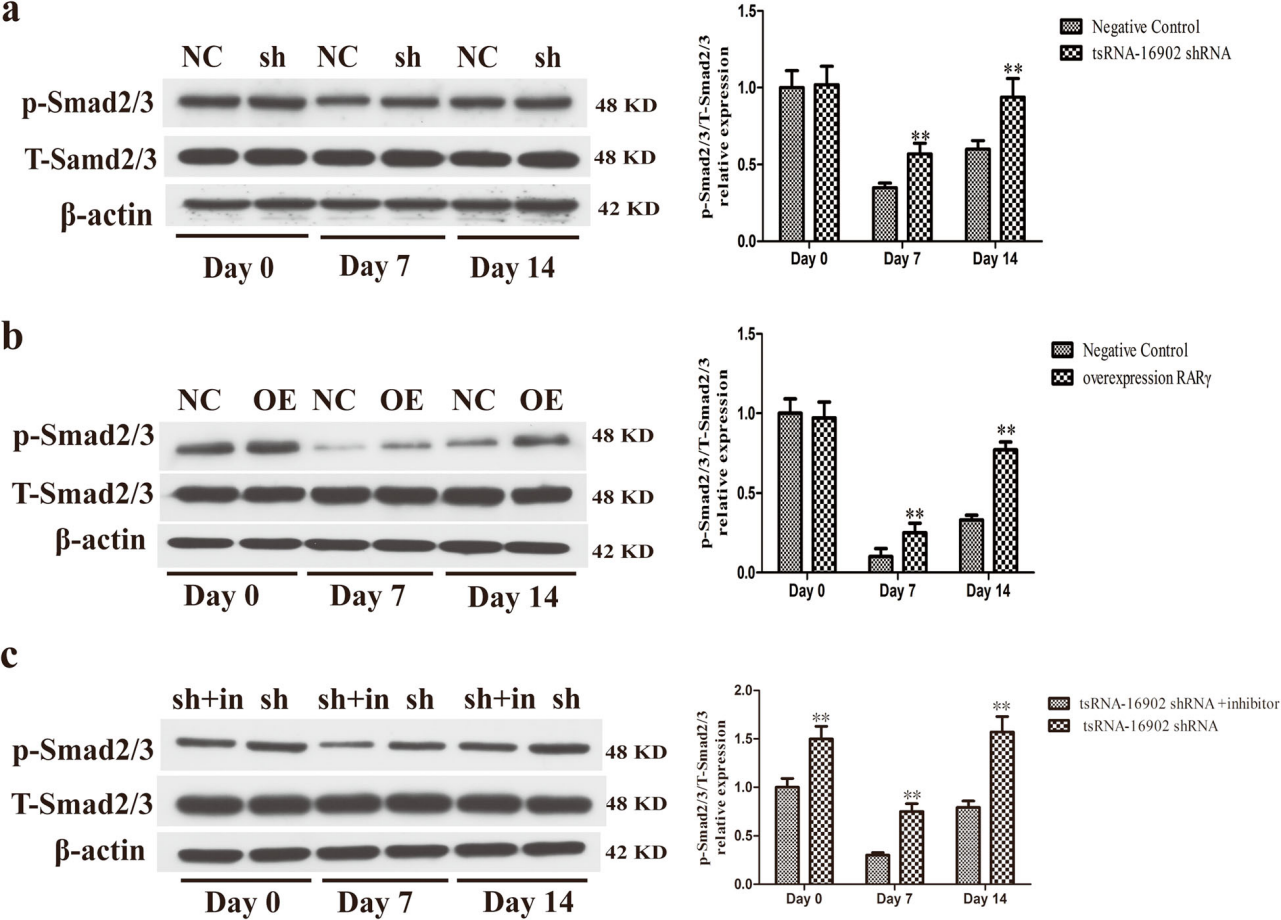
Knockdown of tsRNA-16902 or overexpression of RARγ leads to increased p-Smad2/3 levels, and addition of a Smad2/3 inhibitor reduced p-Smad2/3 levels in the context of adipogenesis as determined by measuring Smad2/3 phosphorylation by Western blotting on days 0, 7 and 14. **a** shRNA-mediated knockdown of tsRNA-16902 alters p-Smad2/3 levels in the context of adipogenic differentiation. **b** RARγ overexpression alters p-Smad2/3 levels in the context of adipogenic differentiation. **c** Smad2/3 inhibition reduced p-Smad2/3 levels during the adipogenic differentiation of hMSCs. Data are expressed as means ± SD (X ± SD, *n* = 3). **P < 0.01 vs. negative controls, respectively (**a**, **b**); **P < 0.01 vs. tsRNA-16902 shRNA + inhibitor (**c**). Note: sh, shRNA; sh+inh, sh + inhibitor. Note: NC, negative control; sh, tsRNA-16902 shRNA; OE, overexpression RARγ; sh+in, tsRNA-16902 shRNA + inhibitor; p-Smad2/3, Smad2/3 phosphorylation; T-Smad2/3, total Smad2/3

We finally explored the ability of altered RARγ and tsRNA-16902 expression to impact Smad2/3 signaling in the context of adipogenesis by measuring Smad2/3 phosphorylation by Western blotting at different time points. These analyses revealed that both tsRNA-16902 knockdown and RARγ overexpression were associated with a significant increase in p-Smad2/3levels (Fig. 8a, b).

### A Smad2/3 inhibitor reverses knockdown tsRNA-16902-mediated hMSC adipogenic differentiation

**Fig. 9:**
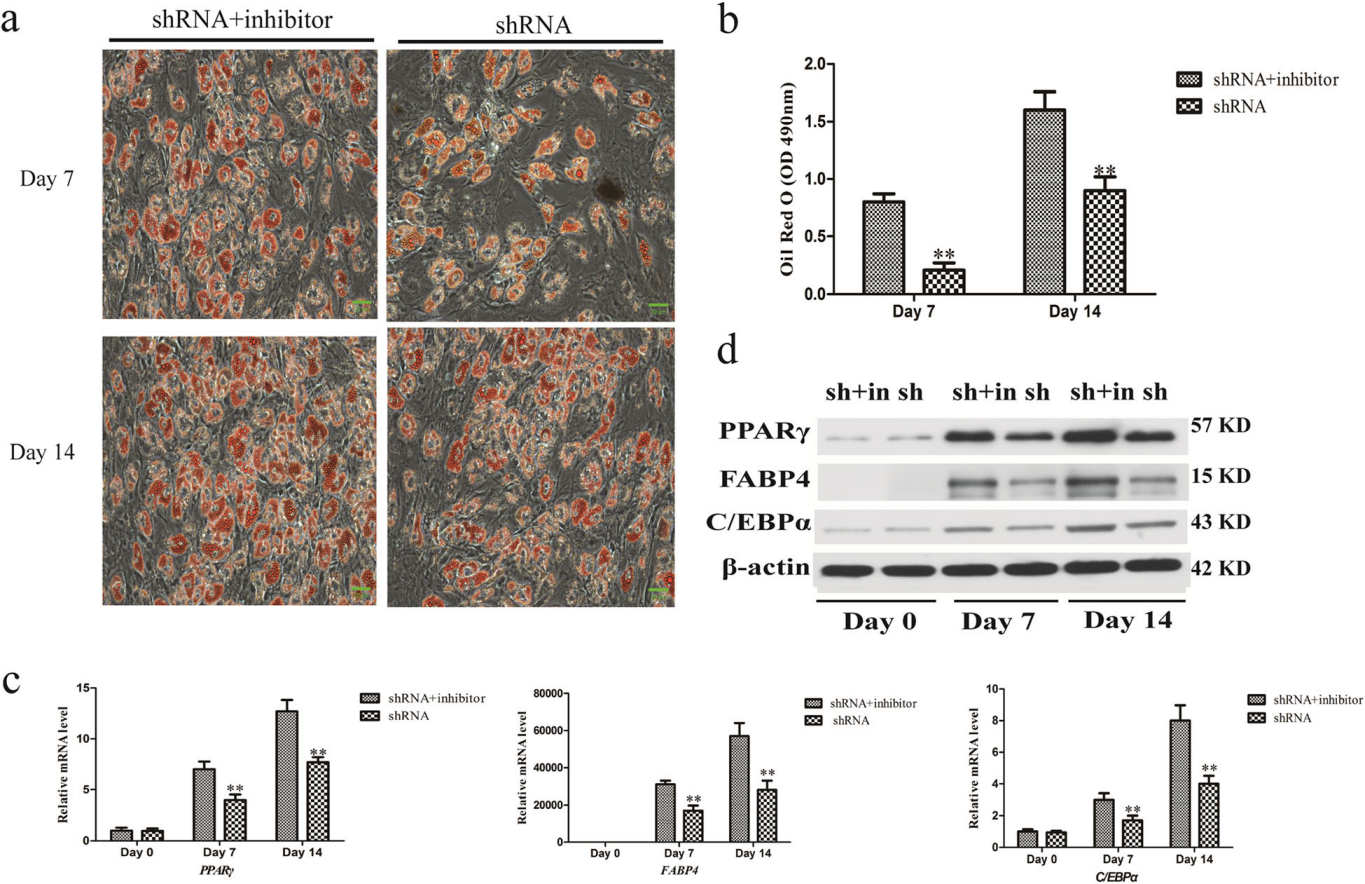
The adipogenic differentiation of hMSCs in which tsRNA-16902 was knocked down and to which an Smad2/3 signaling inhibitor was added. Oil red O staining was employed as a means of assessing lipid accumulation. Scale bar, 50 μm. **a** Following addition of a Smad2/3 signaling inhibitor, the adipogenic differentiation of hMSCs in which tsRNA-16902 was also knocked down was associated with increased lipid accumulation relative to in cells in which tsRNA-16902 was knocked down but to which no inhibitor was added. **b** Differences in Oil red O staining intensity were significant between shRNA + inhibitor and shRNA groups. **c** qRT-PCR was used to compare PPARγ, FABP4, and CEBP/α expression in the different groups. **d** PPARγ, FABP4, and C/EBPαprotein levels were assessed via Western blotting. Data are expressed as means ± SD (X ± SD, *n* = 3). **P < 0.01 vs. shRNA + inhibitor, respectively. Note: sh, tsRNA-16902 shRNA; sh+in, tsRNA-16902 shRNA + inhibitor

We additionally treated cells with a Smad2/3 inhibitor (TP0427736, Selleck) in order to reduce p-Smad2/3 levels in these cells (Fig. 8c), revealing that adipogenic differentiation in hMSCs in which tsRNA-16902 had been knocked down was more robust as compared with that in hMSCs in which tsRNA-16902 had been knocked down in the absence of inhibitor addition (Fig. 9). These results thus further revealed that the Smad2/3 signaling pathway is downstream of tsRNA-16902.

## Discussion

The ability of diverse non-coding RNA molecules to regulate adipogenesis has been increasingly well documented in recent years [34–36]. In addition to better-studied molecules such as miRNAs, lncRNAs, and circRNAs, many other non-coding RNA subtypes, including tsRNAs, have also been identified and shown to be biologically important in diverse contexts [21–25]. The role of tsRNAs in hMSC adipogenic differentiation, however, remains incompletely understood.

In this study, we began by characterizing tsRNA expression profiles during hMSC adipogenic differentiation. This analysis revealed that tsRNAs expressed in these cells were primarily from 17 to 23 and 30–36 nucleotides in length, thus strongly suggesting that these tRNA segments were not the products of random degradation (Fig. 2a). In addition, the majority of 5′-tRNA halves, tRFs-5, 3′-tRNA halves, and tRFs-3 had positions similar to those of the parental tRNAs from which they were derived (Fig. 2b), further confirming that these molecules resulted from specific cleavage events. These findings were thus consistent with previous studies of tsRNA biology [21, 22, 37].

All 6 tsRNA subtypes were evident within our samples, with the majority of these tsRNAs reaching peak expression levels at day 7 of the adipogenic process before declining on days 14 and 21 (Fig. 2c, d). This suggested a potentially pivotal role for these tsRNAs in adipogenesis. We therefore focused on differential tsRNA expression on day 7, as this was both the time point at which most tsRNAs were maximally expressed and a time point known to be critical for pre-adipocyte fate determination in this context [38, 39].

We next assessed patterns of differential tsRNA expression at this day 7 time point (Fig. 3a, b), revealing 6 tsRNAs that were not detectable on day 0 and yet reached peak expression on day 7. This suggested that these tsRNAs may thus be key regulators of adipogenesis, and as such, we next focused on one of these tsRNAs in follow-up experiments. We found that knocking down tsRNA-16902 inhibited hMSC adipogenesis, implying that tsRNA-16902 may promote adipogenic differentiation in this context (Fig. 5). Given the clear impact of tsRNA-16902 knockdown on hMSC adipogenesis, we then studied this tsRNA further.

tsRNAs have been shown to play evolutionarily conserved roles in stress response in eukaryotes [40–42].. tsRNAs have also been shown to play an essential role in the context of cancers, neurological disease, and metabolic disorders [21–25]. In one recent report, researchers found that tRF^GluTTC^ was capable of inhibiting 3T3-L1 preadipocyte differentiation into adipocytes [43]. Interestingly, we identified a distinct tsRNA that was able to modulate hMSC adipogenesis, suggesting that these tsRNAs may function via distinct mechanisms in this context. We also utilized primary hMSCs rather than the 3T3-L1 cell line model, potentially indicating that our results are more physiologically relevant. Other studies have highlighted the ability of tsRNAs to regulate stem cell states in murine embryonic stem cells (mESCs), consistent with our findings [44]. As tsRNAs are a relatively recent area of research, there have been relatively few studies of them to date in vivo [25, 43, 44]. As we utilized human bone marrow mesenchymal stem cells as research subjects in our study, further in vivo validation of our findings will be challenging. However, studies of the adipogenic differentiation of human bone marrow mesenchymal stem cells are common as these cells represent an ideal, and as such, our results are still of great reference value and significance [5, 6, 8]. Although no further in vivo validation experiments were performed in our study, we believe that our data clearly show that tsRNA is likely to play an important role in the adipogenic differentiation process.

How tsRNA-16902 controls hMSC adipogenic differentiation remains to be fully clarified. Several studies to date have proposed distinct mechanisms whereby tsRNAs control gene expression [45–47]. One study suggests that certain tsRNAs may function in a manner similar to miRNAs, guiding Argonaute (Ago) to regulate target gene expression [48]. However, these studies provide contradictory evidence suggesting that tsRNAs and miRNAs function via distinct mechanisms, with tsRNA target sites being present not just in the 3′-UTR but also in the 5′-UTR and CDS regions of target mRNAs [49]. In this study, we identified putative tsRNA-16902 target genes based upon their sequence complementarity, thus identifying RARγ as a putative target of this tsRNA (Fig. 7a).

Retinoic acid (RA) is a key regulator of cell differentiation in many contexts [50–52], functioning so as to control gene expression via activation of the RA receptor (RAR) and retinoid X receptor (RXR) families of nuclear hormone receptors [53]. RARγ activation has been shown to be important for inhibiting PPARγ2 expression in 3T3-L1 adipocyte differentiation [54]. This thus suggests that RARγ may be an important regulator of adipogenesis. Consistent with this, we found that RARγ overexpression impaired hMSC adipogenesis (Fig. 6), thus confirming its relevance in this process. We further found that the expression of tsRNA-16902 and RARγ were negatively correlated with one another (Fig. 7b). Using a luciferase reporter assay, we then further confirmed that RARγ was a direct target of tsRNA-16902 (Fig. 7c).

**Fig. 10:**
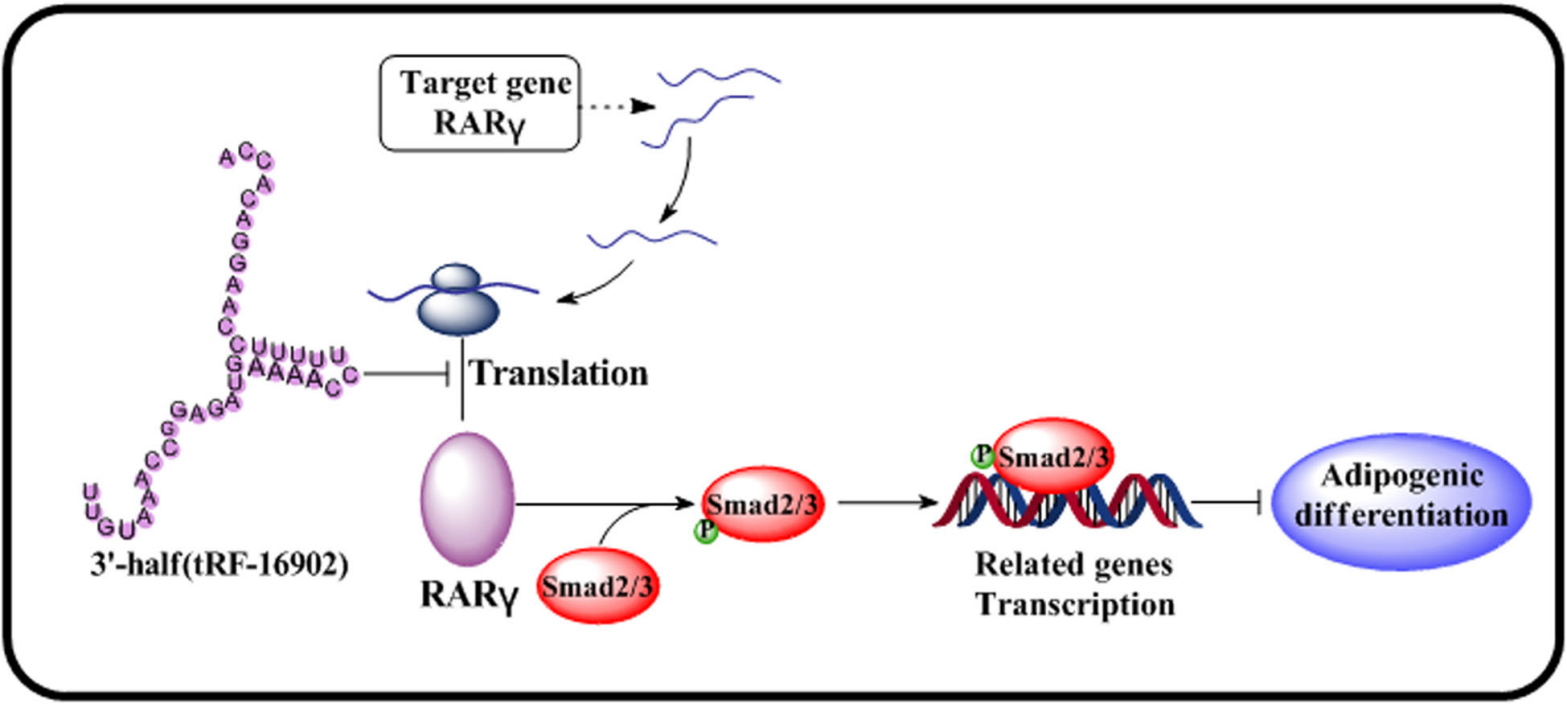
A potential model wherein tsRNA-16902 controls hMSC adipogenesis by targeting of RARγ via the Smad2/3 signaling pathway

It is well known that there are many signaling pathways that affect the adipogenic differentiation of hMSCs. Selecting the appropriate signaling pathway downstream of RARγ is very important. The Smad2/3 signaling pathway has recently been shown to be central to the regulation of adipogenesis [55–57]. In addition, this signaling pathway involved in non-coding RNA has also been shown to play an essential role in the context of cancers, cell differentiation, and migration [58–60]. Interestingly, the relevant signaling pathways whereby tsRNAs modulate cellular functionality have only been analyzed via bioinformatics approaches [61, 62], suggesting that much research remains to be conducted in this area. Therefore, the relationship between tsRNA-16902 and Smad2/3 signaling pathway was a topic of interest in the present study. We found that both tsRNA-16902 knockdown and RARγ overexpression resulted in enhanced Smad2/3 phosphorylation in hMSCs (Fig. 8a, b). Furthermore, by adding a Smad2/3 inhibitor to suppress p-Smad2/3 levels (Fig. 8c), we were able to demonstrate that the adipogenic differentiation of hMSCs in which tsRNA-16902 was also knocked down was more robust than that in cells in which tsRNA-16902 was knocked down but to no inhibitor was added (Fig. 9). These results thus further confirmed that the Smad2/3 signaling pathway is downstream of RARγ, with tsRNA-16902 controlling the activation of this pathway. Based on these findings, we therefore propose a model wherein tsRNA-16902 can regulate hMSC adipogenesis by targeting RARγ via the Smad2/3 signaling (Fig. 10).

## Conclusions

In summary, using a high-throughput sequencing analysis, we assessed patterns of tsRNA expression during hMSC adipogenesis. This approach led us to identify tsRNA-16902 as a novel and important regulator of this process. These results may thus offer new opportunities for the treatment of obesity.

## Supplementary information

**Additional file 1.**

**Additional file 2.**

**Additional file 3.**

## Data Availability

All data generated and/or analyzed during this study are included in this published article.

## Abbreviations

tsRNAs: tRNA-derived small RNAs
hMSCs: Human bone marrow mesenchymal stem cells
RARγ: Retinoic acid receptor γ
PPARγ: Peroxisome proliferator-activated receptor γ
C/EBPα: CCAAT enhancer binding protein α
FABP4: Fatty Acid Binding Protein-4
qRT-PCR: Quantitative real-time polymerase chain reaction
UTR: Untranslated region
