# Supplementary figures and images for: A novel tsRNA-16902 regulating the adipogenic differentiation of human bone marrow mesenchymal stem cells
Source: Stem Cell Res Ther. 2020 Aug 24;11:365. doi: 10.1186/s13287-020-01882-6 (PMC7444066; doi:10.1186/s13287-020-01882-6)

**FIGURE S1:** Sequencing data analysis flowchart.

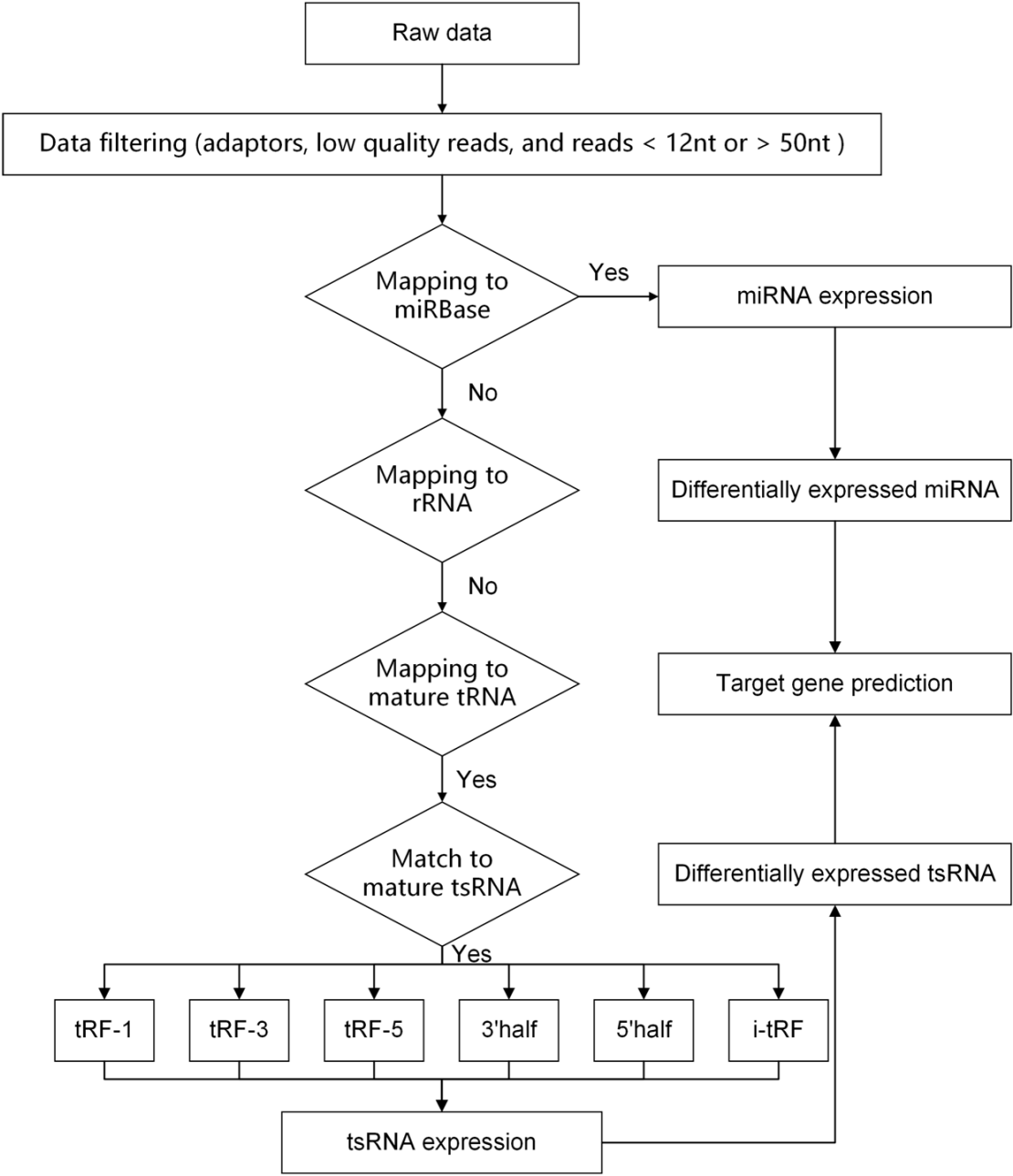

Supplement: Supplementary file 2 — Additional file 2. [file 13287_2020_1882_MOESM2_ESM.pdf]
