# Supplementary material for: A novel tsRNA-16902 regulating the adipogenic differentiation of human bone marrow mesenchymal stem cells
Source: Stem Cell Res Ther. 2020 Aug 24;11:365. doi: 10.1186/s13287-020-01882-6 (PMC7444066; doi:10.1186/s13287-020-01882-6)
Supplement: Supplementary file 3 — Additional file 3. [file 13287_2020_1882_MOESM3_ESM.docx]

| Gene symbol | Forward primer | Reverse primer | Length (bp) |
| --- | --- | --- | --- |
| *PPARγ* | 5'- GGGATGTCTCATAATGCCATCAG- 3' | 5'-GCCCTCGCCTTTGCTTTG-3' | 97 |
| *CEBP/α* | 5'-CCAAGAAGTCGGTGGACAAGAAC-3' | 5'-CACCTTCTGCTGCGTCTCCA-3' | 122 |
| *FABP4* | 5-'GGATGATAAACTGGTGGTGGAATG-3' | 5'- CAGAATGTTGTAGAGTTCAATGCGA -3' | 123 |
| *STC2* | 5-'TGTGGCGTGTTTGAATGTTT-3 | 5-'CACAGGTCGTGCTTGAGGTA-3 | 245 |
| *β-actin* | 5'-GCGAGAAGATGACCCAGATCATGT-3' | 5'-TACCCCTCGTAGATGGGCACA-3' | 160 |

**Table S1**: Primers used in this study forβ-actin, PPARγ, C/EBPα, FABP4, and STC2.
