# Supplementary material for: A novel tsRNA-16902 regulating the adipogenic differentiation of human bone marrow mesenchymal stem cells
Source: Stem Cell Res Ther. 2020 Aug 24;11:365. doi: 10.1186/s13287-020-01882-6 (PMC7444066; doi:10.1186/s13287-020-01882-6)
Supplement: Supplementary file 1 — Additional file 1. [file 13287_2020_1882_MOESM1_ESM.docx]

Dentification of hMSCs via **flow cytometry**

We thank the Reviewer for this valuable suggestion. In this revision, we have added cell Lot No of three donors that was initially ommitted and have added flow cytometry data and the demographics corresponding to these hMSCs in the supplementary materials as follow.

The results are shown below, and the manuscript has been updated accordingly.

**Lot No: 150724I31**

(Age: 26; Body mass index: 21.7; Gender:male; Ethnicity: Chinese)

**Lot No: 160202I31**

(Age: 28; Body mass index: 20.9; Gender:female; Ethnicity: Chinese)

**Lot No: 161125R41**

(Age: 25; Body mass index:22.3; Gender:male; Ethnicity:Chinese)

Using the following antibodies:

FITC Mouse Anti-Human CD90( Catalog number:561969) ;

PE Mouse Anti-Human CD73( Catalog number:561014);

PE Mouse Anti-Human CD105( Catalog number:560839);

PE Mouse Anti-Human CD34( Catalog number:560941);

PE Mouse Anti-Human CD11b( Catalog number:561001);

FITC Mouse Anti-Human CD45( Catalog number:560976) ;

FITC Mouse Anti-Human CD19( Catalog number:560994) ;

FITC Mouse Anti-Human HLA-DR( Catalog number:560944);

All antibodies were purchased from BD Biosciences.
